# Supplementary material for: Spatiotemporal dynamics of multidrug resistant bacteria on intensive care unit surfaces
Source: Nat Commun. 2019 Oct 8;10:4569. doi: 10.1038/s41467-019-12563-1 (PMC6783542; doi:10.1038/s41467-019-12563-1)
Supplement: Supplementary file 1 — Supplementary Information [file 41467_2019_12563_MOESM1_ESM.pdf]

Supplementary Information

Spatiotemporal dynamics of multidrug resistant bacteria on intensive care unit surfaces

Alaric W. D'Souza[1]*, Robert F. Potter[1]*, Meghan Wallace[2], Angela Shupe[2], Sanket Patel[1,2], Xiaoqing Sun[1,2], Danish Gul[3], Jennie H. Kwon[4], Saadia Andleeb[3]**, Carey-Ann D. Burnham[2,4,5,6]**, Gautam Dantas[1,2,5,7]**

*These authors contributed equally to this work.

**Corresponding Authors

(Saadia Andleeb: saadiamarwat@yahoo.com, Carey-Ann D. Burnham: cburnham@wustl.edu, Gautam Dantas: dantas@wustl.edu)

[1]The Edison Family Center for Genome Sciences and Systems Biology, Washington University School of Medicine, St. Louis, MO, USA

[2]Department of Pathology and Immunology, Washington University School of Medicine, St. Louis, MO, USA

[3]Atta ur Rahman School of Applied Biosciences, National University of Sciences and Technology Islamabad, Pakistan

[4]Department of Medicine, Washington University School of Medicine, St. Louis, MO, USA

[5]Department of Molecular Microbiology, Washington University School of Medicine, St. Louis, MO, USA

[6]Departments of Pediatrics, Washington University School of Medicine, St. Louis, MO, USA

[7]Department of Biomedical Engineering, Washington University in St. Louis, St. Louis, MO, USA

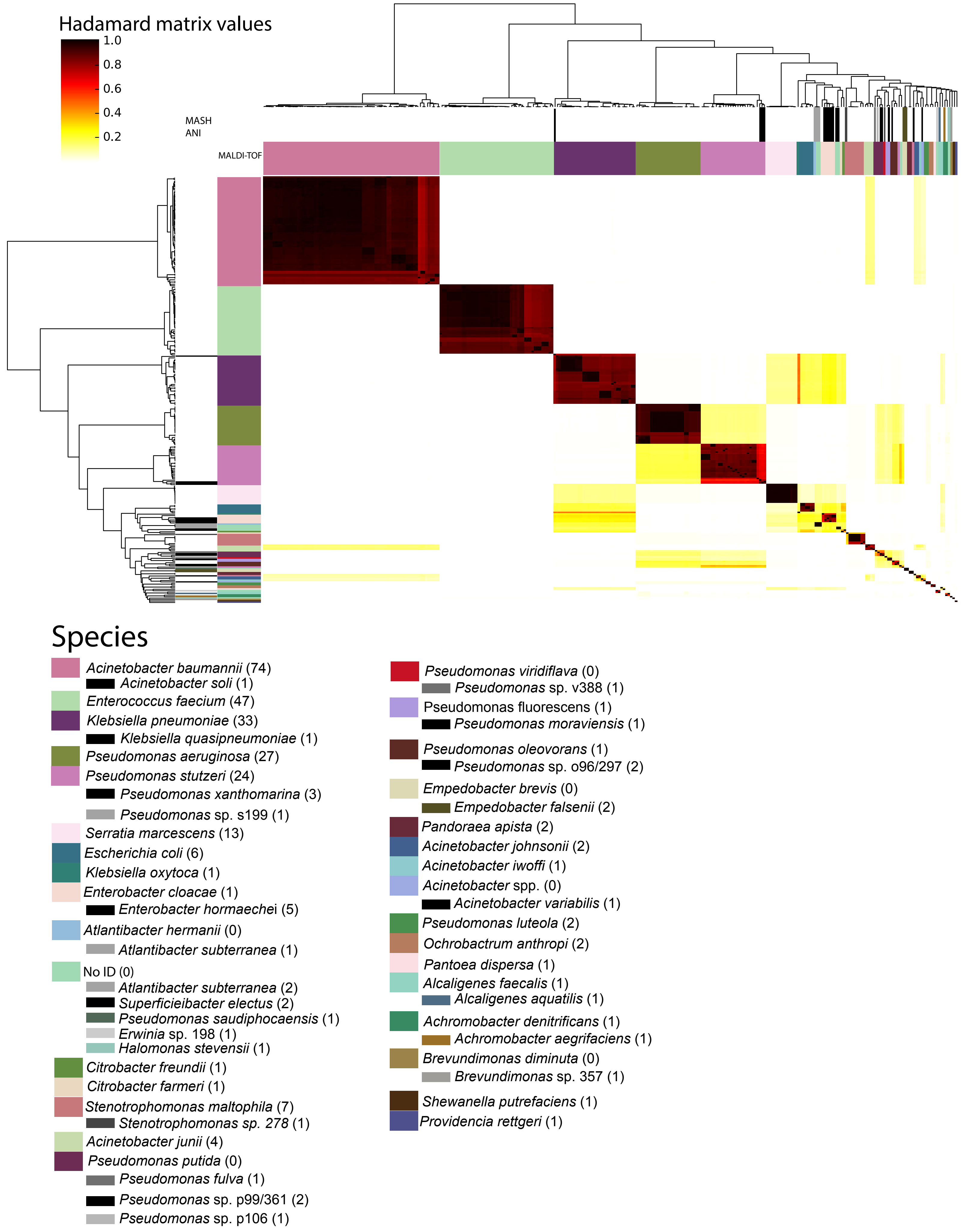

Hadamard matrix values
1.0
0.8
0.6
0.4
0.2
MASH
ANI
MALDI-TOF
Species
Acinetobacter baumannii (74)
Acinetobacter soli (1)
Enterococcus faecium (47)
Klebsiella pneumoniae (33)
Klebsiella quasipneumoniae (1)
Pseudomonas aeruginosa (27)
Pseudomonas stutzeri (24)
Pseudomonas xanthomarina (3)
Pseudomonas sp. s199 (1)
Serratia marcescens (13)
Escherichia coli (6)
Klebsiella oxytoca (1)
Enterobacter cloacae (1)
Enterobacter hormaechei (5)
Atlantibacter hermanii (0)
Atlantibacter subterranea (1)
No ID (0)
Atlantibacter subterranea (2)
Superficieibacter electus (2)
Pseudomonas saudiphocaensis (1)
Erwinia sp. 198 (1)
Halomonas stevensii (1)
Citrobacter freundii (1)
Citrobacter farmeri (1)
Stenotrophomonas maltophila (7)
Stenotrophomonas sp. 278 (1)
Acinetobacter junii (4)
Pseudomonas putida (0)
Pseudomonas fulva (1)
Pseudomonas sp. p99/361 (2)
Pseudomonas sp. p106 (1)
Pseudomonas viridiflava (0)
Pseudomonas sp. v388 (1)
Pseudomonas fluorescens (1)
Pseudomonas moraviensis (1)
Pseudomonas oleovorans (1)
Pseudomonas sp. o96/297 (2)
Empedobacter brevis (0)
Empedobacter falsenii (2)
Pandoraea apista (2)
Acinetobacter johnsonii (2)
Acinetobacter iwoffi (1)
Acinetobacter spp. (0)
Acinetobacter variabilis (1)
Pseudomonas luteola (2)
Ochrobactrum anthropi (2)
Pantoea dispersa (1)
Alcaligenes faecalis (1)
Alcaligenes aquatilis (1)
Achromobacter denitrificans (1)
Achromobacter aegrifaciens (1)
Brevundimonas diminuta (0)
Brevundimonas sp. 357 (1)
Shewanella putrefaciens (1)
Providencia rettgeri (1)

Supplementary Figure 1: Taxonomic identification by WGS gives higher resolution than MALDI-TOF MS

Pairwise average nucleotide identity (ANI) between all PAK-H isolates. Axes of this graph are hierarchically clustered and the MALDI-TOF MS identifications are shown as colored annotations next to the heatmap. MASH ANI species identifications to type strains are shown as colored annotations where they differed from MALDI-TOF MS annotations. Source data is provided in the source data file.

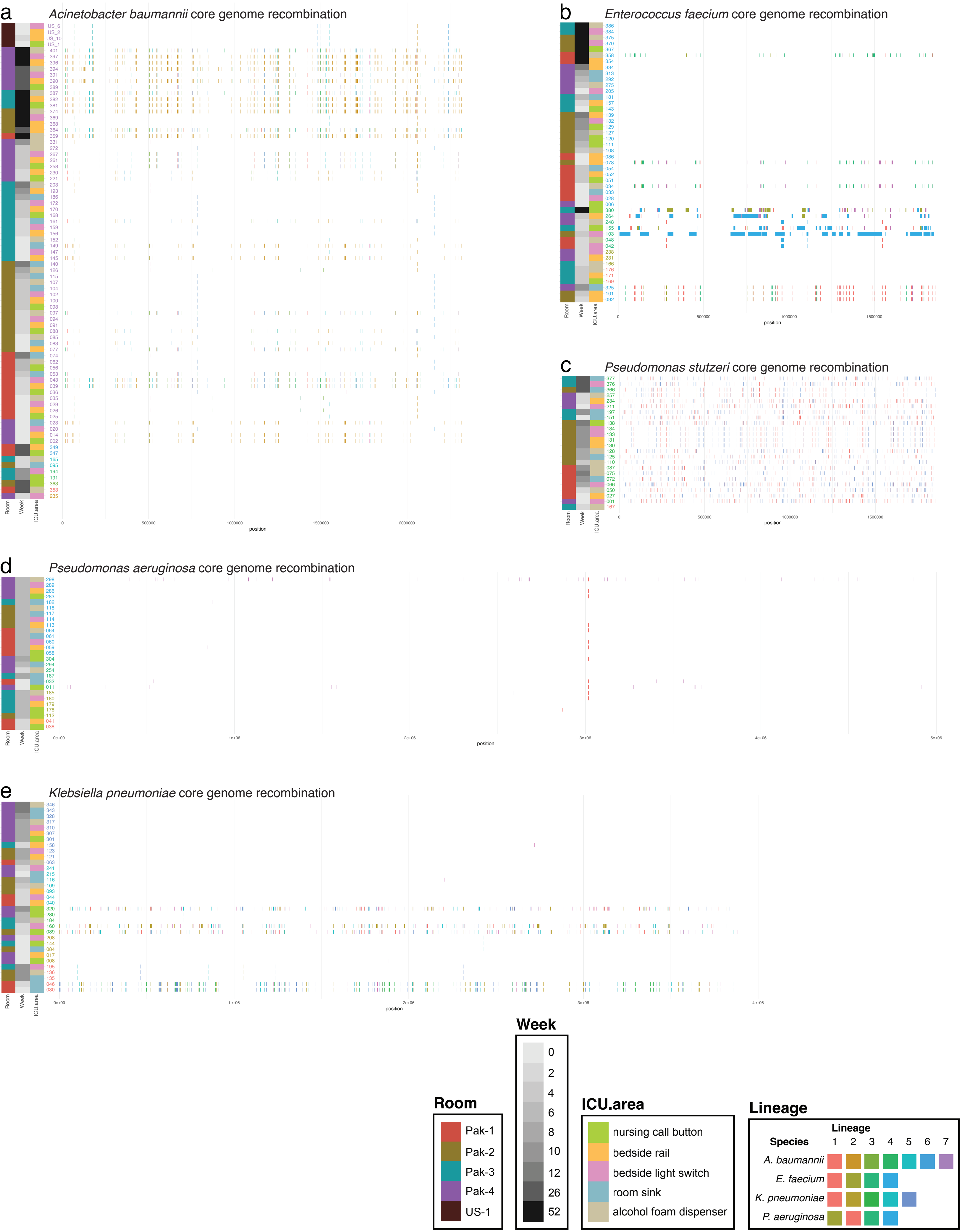

a
Acinetobacter baumannii core genome recombination
Room
Week
ICU.area
position
0
500000
1000000
1500000
2000000
b
Enterococcus faecium core genome recombination
Room
Week
ICU.area
position
0
500000
1000000
1500000
c
Pseudomonas stutzeri core genome recombination
Room
Week
ICU.area
position
0
500000
1000000
1500000
d
Pseudomonas aeruginosa core genome recombination
Room
Week
ICU.area
position
0e+00
1e+06
2e+06
3e+06
4e+06
5e+06
e
Klebsiella pneumoniae core genome recombination
Room
Week
ICU.area
position
0e+00
1e+06
2e+06
3e+06
4e+06
Room
Pak-1
Pak-2
Pak-3
Pak-4
US-1
Week
0
2
4
6
8
10
12
26
52
ICU.area
nursing call button
bedside rail
bedside light switch
room sink
alcohol foam dispenser
Lineage
Lineage
Species
1 2 3 4 5 6 7
A. baumannii
E. faecium
K. pneumoniae
P. aeruginosa

Supplementary Figure 2: Recombinant core genome positions for major species

Core-genome alignments with recombinant positions for **(a)** *A. baumannii*, **(b)** *E. faecium*, **(c)** *P. stutzeri*, **(d)** *P. aeruginosa*, **(e)** *K. pneumoniae*. The x-axis for each plot is the position in the core genome and each row is an isolate. Vertical bars in the plot show where recombination is predicted, and the colors correspond to the lineage of origin. Isolate numbers are colored by hierBAPS lineage and each isolate is annotated on the left with room, week, and surface information. Source data for all panels is provided in the source data file.

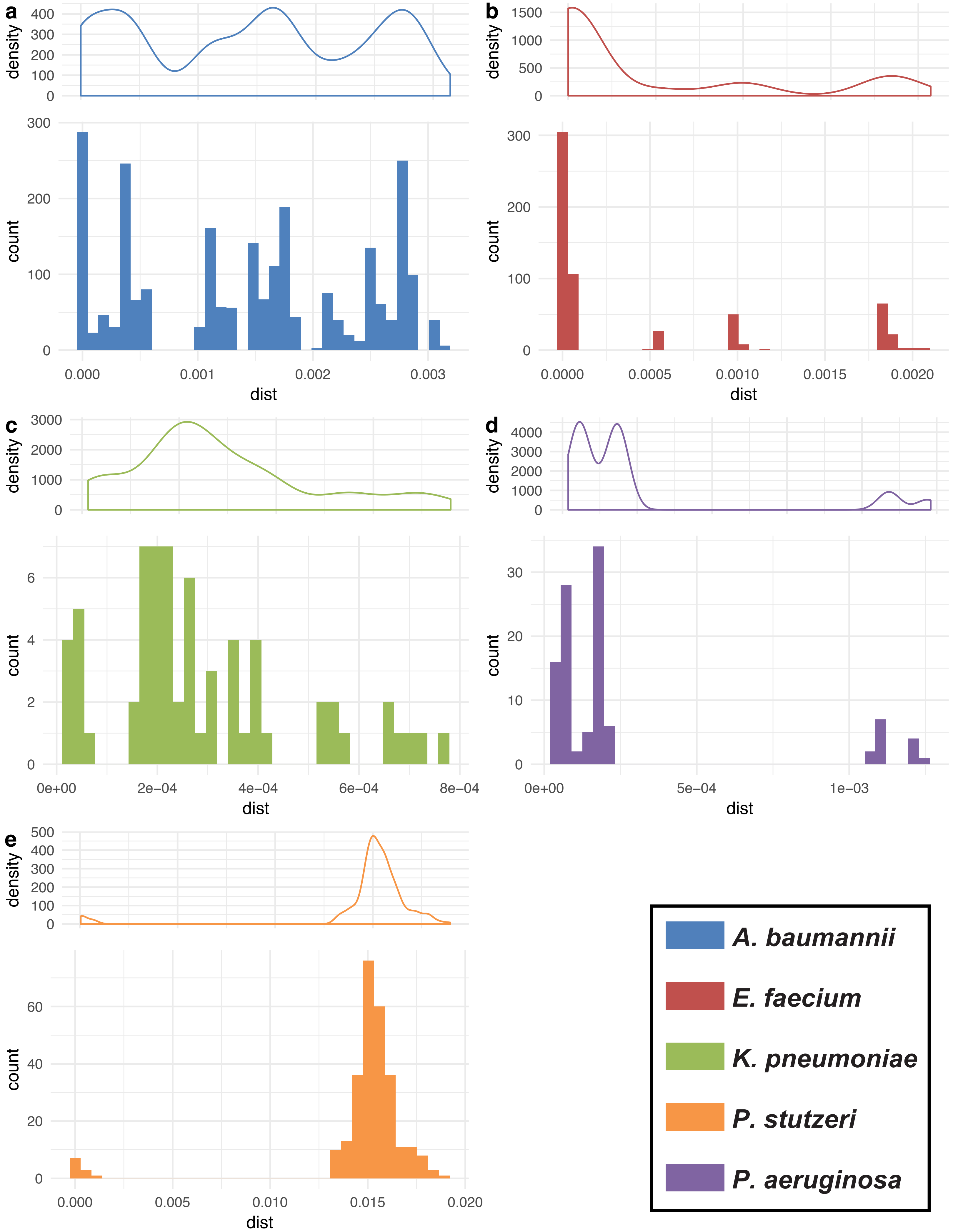

a
b
c
d
e
density
count
dist
A. baumannii
E. faecium
K. pneumoniae
P. stutzeri
P. aeruginosa

Supplementary Figure 3: Histograms of pairwise core genome SNP distances for major species

Distribution of pairwise SNP distances between isolates for **(a)** *A. baumannii*, **(b)** *E. faecium*, **(c)** *K. pneumoniae*, **(d)** *P. aeruginosa*, **(e)** *P. stutzeri*. The x-axis is the ratio of number of pairwise SNPs to core-genome length and the y-axis is counts for the histograms and density for the density plots. Source data for all panels is provided in the source data file.

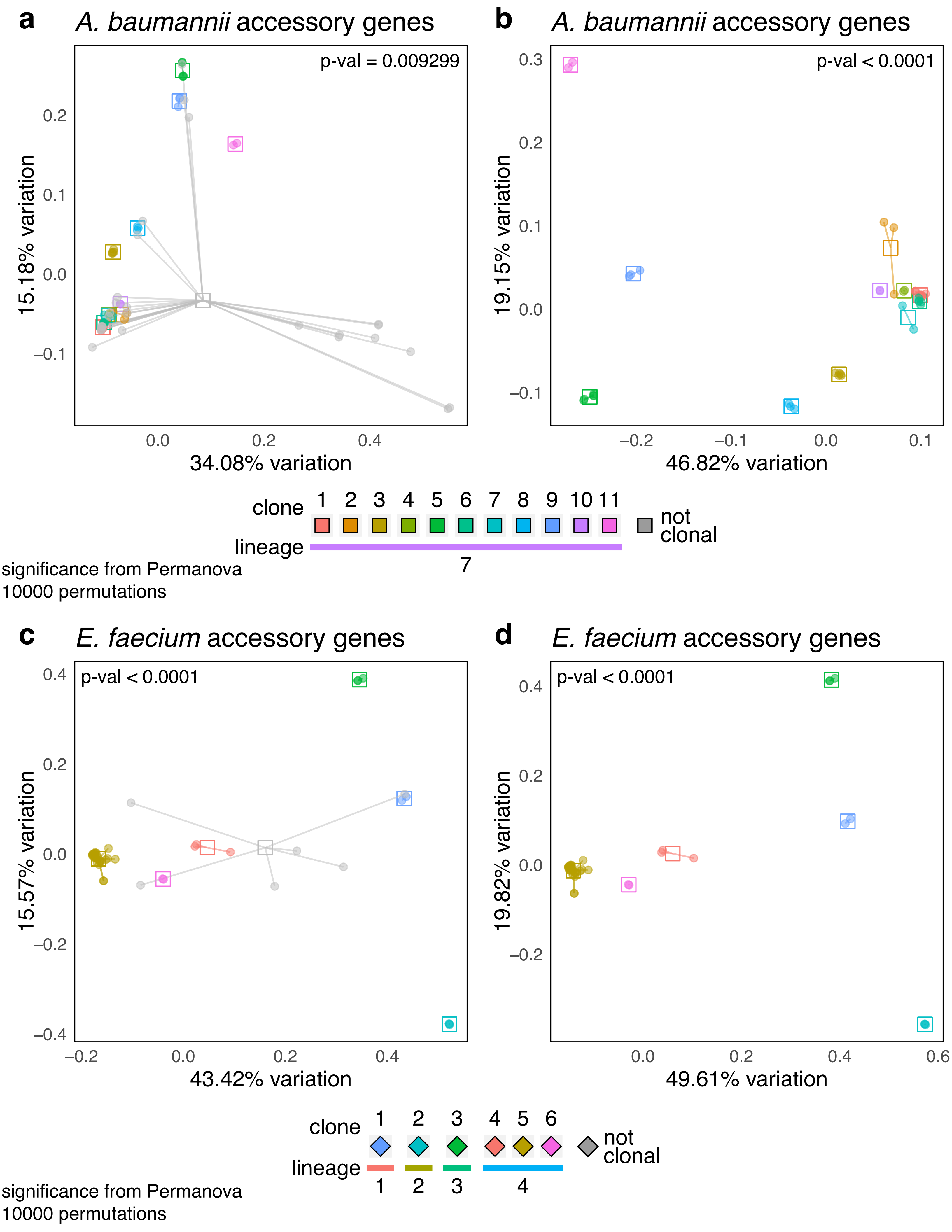
a
A. baumannii accessory genes
p-val = 0.009299
15.18% variation
34.08% variation
b
A. baumannii accessory genes
p-val < 0.0001
19.15% variation
46.82% variation
clone
1 2 3 4 5 6 7 8 9 10 11
not clonal
lineage
7
significance from Permanova
10000 permutations
c
E. faecium accessory genes
p-val < 0.0001
15.57% variation
43.42% variation
d
E. faecium accessory genes
p-val < 0.0001
19.82% variation
49.61% variation
clone
1 2 3 4 5 6
not clonal
lineage
1 2 3 4
significance from Permanova
10000 permutations

Supplementary Figure 4: Accessory genome ordination of *A. baumannii* and *E. faecium*

Principal coordinates analysis of accessory genome for **(a)** *A. baumannii* with non-clonal isolates included, **(b)** *A. baumannii* without non-clonal isolates, **(c)** *E. faecium* with non-clonal isolates included, **(d)** *E. faecium* without non-clonal isolates. Samples for each specific isolate are filled circles and centers of gravity for each clonal group are open squares. Lines connect samples to their centers of gravity. PERMANOVA was used to determine was used compare clonal groups' centroids and dispersion. Source data for all panels is provided in the source data file.

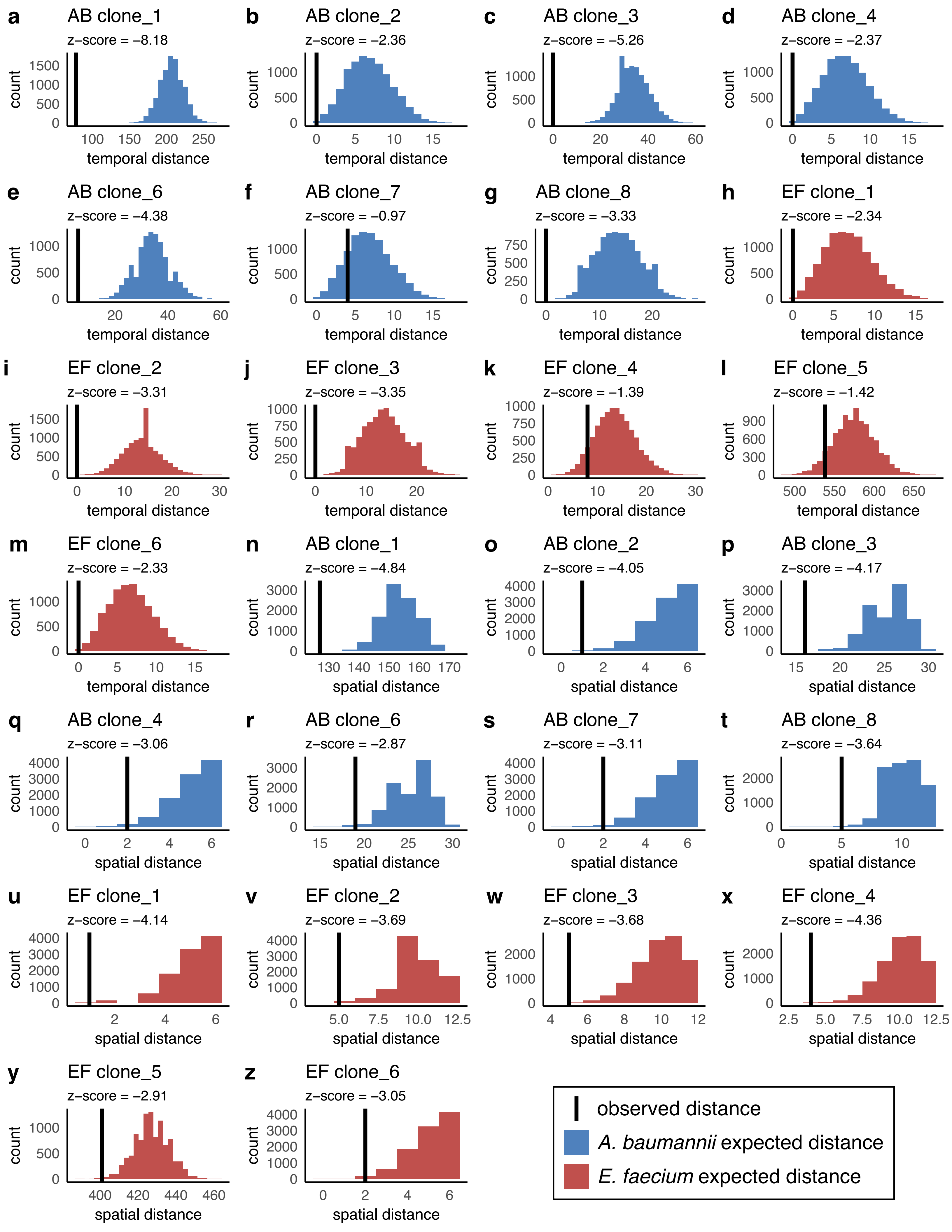

a AB clone_1 z-score = −8.18
b AB clone_2 z-score = −2.36
c AB clone_3 z-score = −5.26
d AB clone_4 z-score = −2.37
e AB clone_6 z-score = −4.38
f AB clone_7 z-score = −0.97
g AB clone_8 z-score = −3.33
h EF clone_1 z-score = −2.34
i EF clone_2 z-score = −3.31
j EF clone_3 z-score = −3.35
k EF clone_4 z-score = −1.39
l EF clone_5 z-score = −1.42
m EF clone_6 z-score = −2.33
n AB clone_1 z-score = −4.84
o AB clone_2 z-score = −4.05
p AB clone_3 z-score = −4.17
q AB clone_4 z-score = −3.06
r AB clone_6 z-score = −2.87
s AB clone_7 z-score = −3.11
t AB clone_8 z-score = −3.64
u EF clone_1 z-score = −4.14
v EF clone_2 z-score = −3.69
w EF clone_3 z-score = −3.68
x EF clone_4 z-score = −4.36
y EF clone_5 z-score = −2.91
z EF clone_6 z-score = −3.05
count
temporal distance
spatial distance
observed distance
A. baumannii expected distance
E. faecium expected distance

Supplementary Figure 5: Spatial and temporal linkage of core genome SNP identified clonal groups

Temporal and spatial linkage for *A. baumannii* and *E. faecium* clones during the first 3 months of collection. Temporal distances are calculated as +1 for every 2 week span separating isolate collections. Spatial distances are given as +0 if isolates were collected from the same surface and room, +1 if they were collected from the same room, but different surfaces, and +2 if they were collected from different rooms. Expected distance distributions for *A. baumannii* are shown in blue and expected distance distributions for *E. faecium* are shown in red. Observed distances are shown as vertical black lines. Z-scores for the observed values compared to the expected distribution are given for each graph. **(a-g)** Temporal distance distributions for *A. baumannii* clones. **(h-m)** Temporal distance distributions for *E. faecium* clones. **(n-t)** Spatial distance distributions for *A. baumannii* clones. **(u-z)** Spatial distance distributions for *E. faecium* clones. Source data for all panels is provided in the source data file.

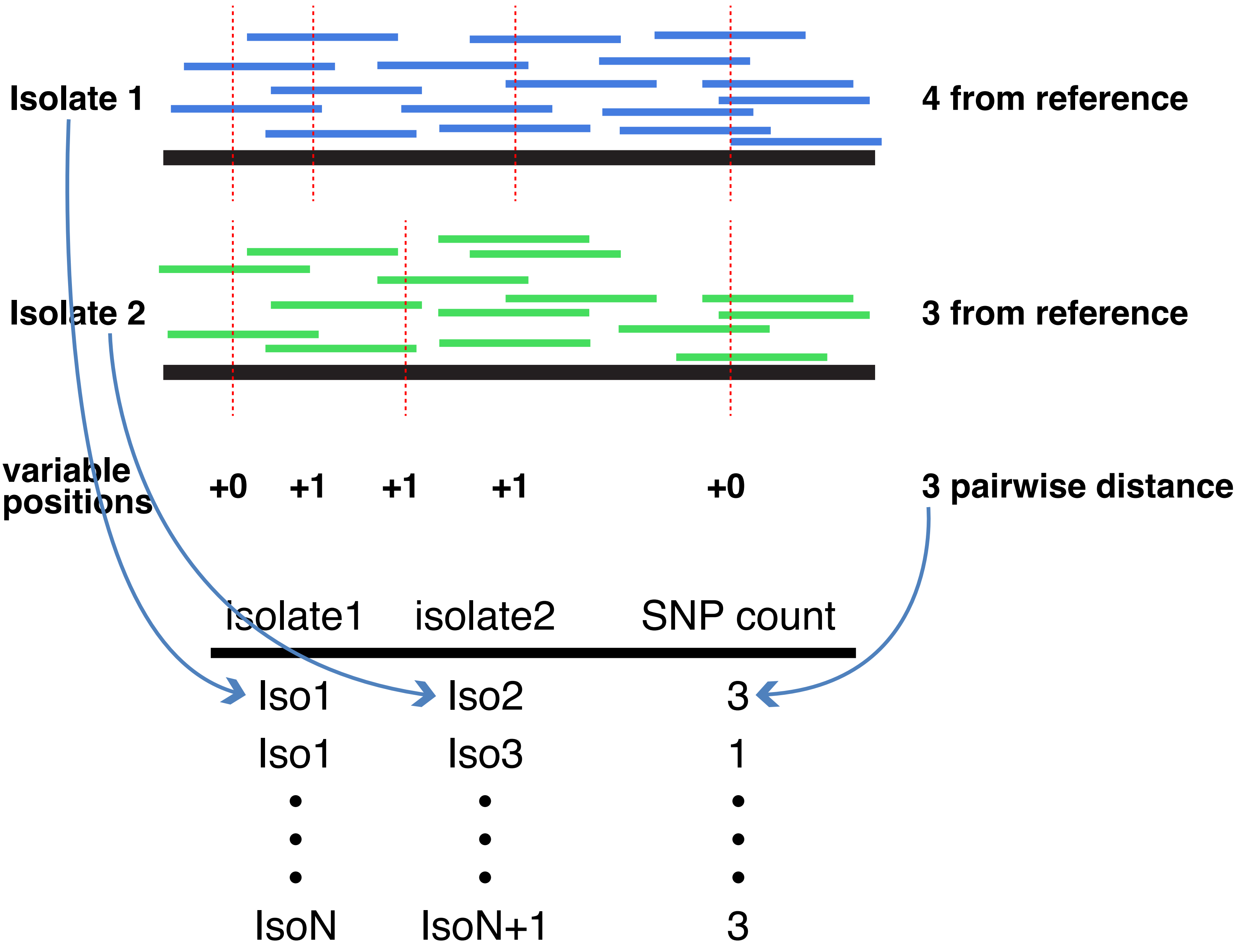
Isolate 1
4 from reference
Isolate 2
3 from reference
variable positions
+0
+1
+1
+1
+0
3 pairwise distance
isolate1
isolate2
SNP count
Iso1
Iso2
3
Iso1
Iso3
1
IsoN
IsoN+1
3

Supplementary Figure 6: Identification of pairwise variant distances from reference read mapping

Dark black horizontal lines represent reference genomes and colored short horizontal lines represent mapped short reads. Vertical dashed red lines represent variant positions from the reference. Every position is checked for differences from the reference and from all other isolates.

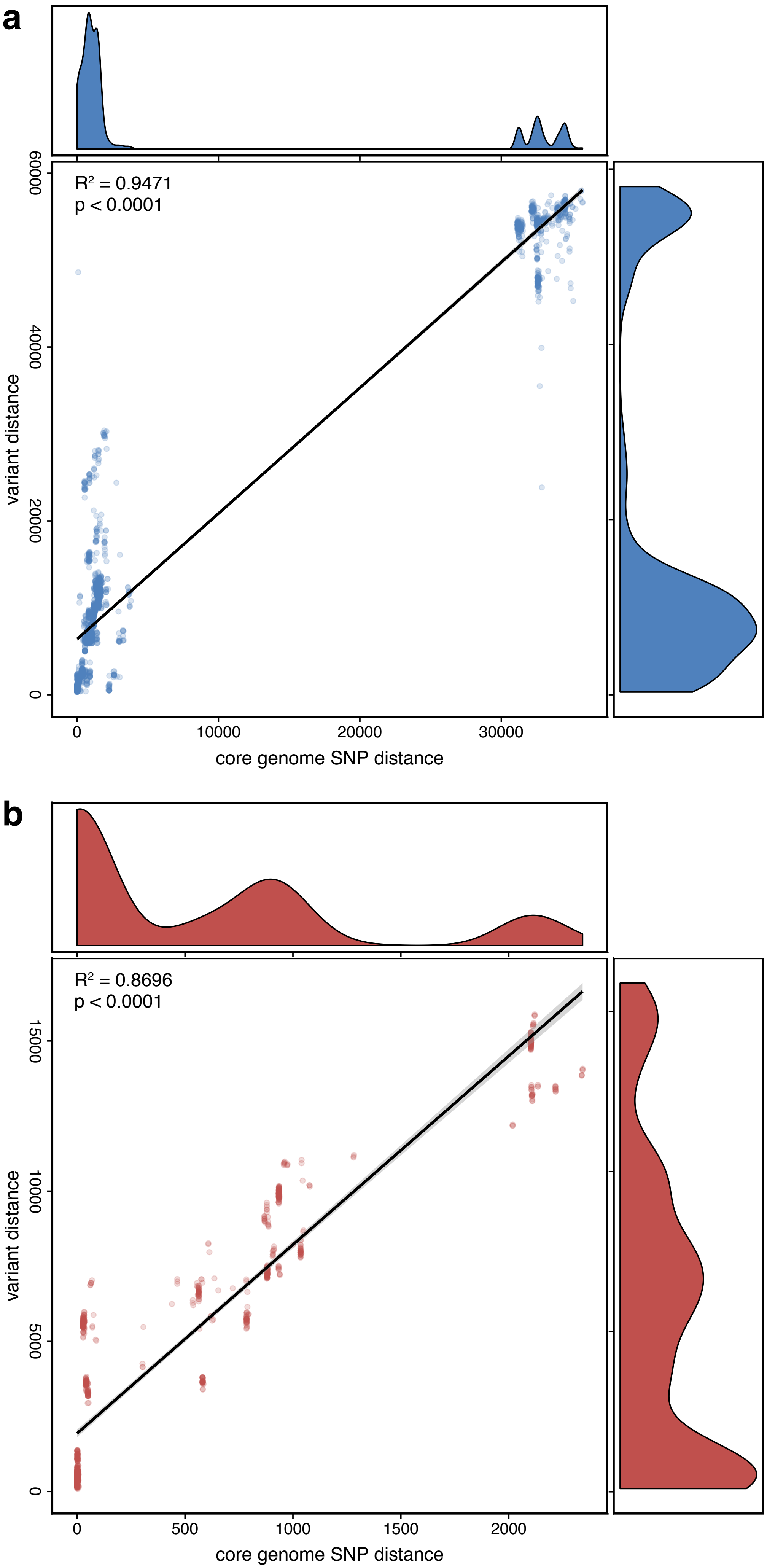
a
R² = 0.9471
p < 0.0001
variant distance
0
20000
40000
60000
0
10000
20000
30000
core genome SNP distance
b
R² = 0.8696
p < 0.0001
variant distance
0
5000
10000
15000
0
500
1000
1500
2000
core genome SNP distance

Supplementary Figure 7: Correlation between core genome SNP and variant distances

Comparison of pairwise core genome SNP distances to pairwise variant distances for **(a)** *A. baumannii* in blue and **(b)** *E. faecium* in red. Each point shows the distance a pairwise comparison of two isolates with the x-axis showing the distance by core genome SNPs and the y-axis showing the distance by variants called by mapping quality filtered reads to the type strain. The density of the points for the core genome SNP and variant axis are shown above and to the right of the scatterplots. The black line is the prediction given by the linear model (*variant distance ~ SNP distance*) and the shaded grey area around the line is the 95% confidence interval for the correlation. The adjusted R^2 coefficient of determination and the p-value are given on the respective plots. Source data for all panels is provided in the source data file.

For each cutoff:

1. filter pairwise distance table by cutoff

isolate1	isolate2	variant dist
~~Iso1~~	~~Iso2~~	~~3~~
Iso1	Iso3	1
⋮	⋮	⋮
~~IsoN~~	~~IsoN+1~~	~~3~~

2. find all maximally connected cliques

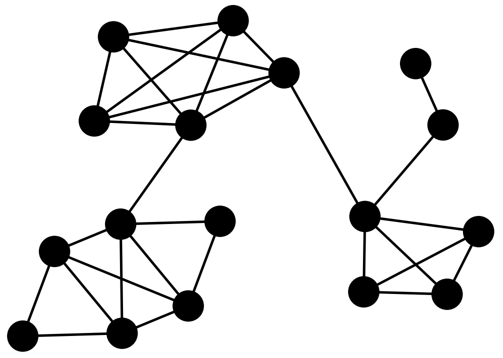

3. sort cliques by highest minimum edgeweight

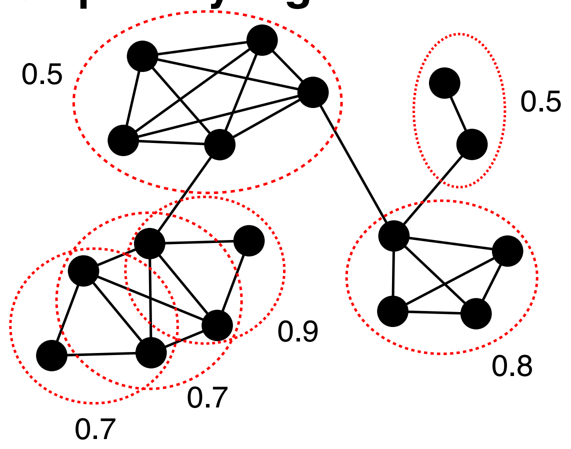

4. remake graph without strongest clique

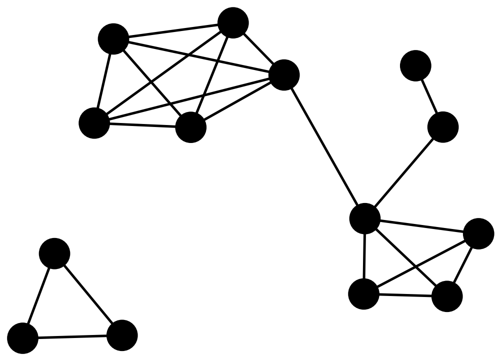

5. recurse on steps 3 and 4

Supplementary Figure 8: Identification of cliques at given cutoff value

In step 1, the pairwise distance table is filtered by the cutoff value. In step 2, this filtered table is then used to generate a network. In step 3, the network is searched for fully connected cliques. The weakest connection in each fully connected clique is identified and cliques are sorted in descending order based on their weakest connection. In step 4, the clique with the highest value weakest connection is stored and its associated nodes are removed from the filtered table. The network is then regenerated without the nodes. For step 5, steps 3 and 4are repeated until all maximally connected cliques are identified.

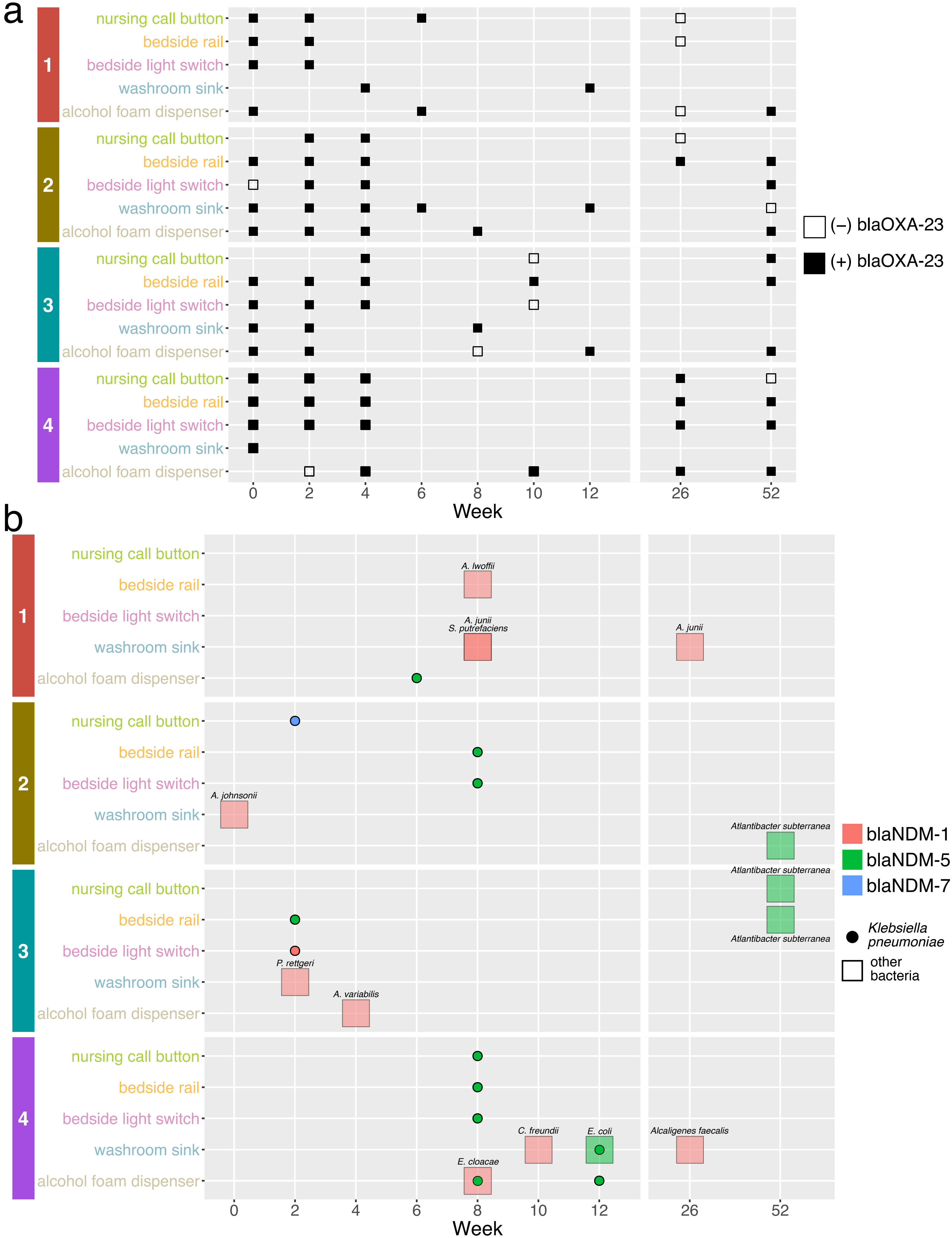
a
1
2
3
4
nursing call button
bedside rail
bedside light switch
washroom sink
alcohol foam dispenser
(–) blaOXA-23
(+) blaOXA-23
0
2
4
6
8
10
12
26
52
Week
b
A. lwoffii
A. junii
S. putrefaciens
A. junii
A. johnsonii
Atlantibacter subterranea
Atlantibacter subterranea
Atlantibacter subterranea
P. rettgeri
A. variabilis
C. freundii
E. coli
Alcaligenes faecalis
E. cloacae
blaNDM-1
blaNDM-5
blaNDM-7
Klebsiella pneumoniae
other bacteria
Week

Supplementary Figure 9: Spatial and temporal organization of high-risk resistance genes

(a) Overview of *bla*$_{\text{OXA-23}}$ on PAK-H surfaces. Each horizontal grey panel represents a PAK-H room. Within each room, the horizontal gridded white lines are the five sampled surfaces. Each vertical white line is one of the collection weeks. Places where the horizontal and vertical white lines intersect represent a sampling. Filled black squares are *A. baumannii* isolates positive for *bla*$_{\text{OXA-23}}$ and unfilled black squares are *A. baumannii* isolates negative for *bla*$_{\text{OA-23}}$.

(b) Overview of *bla*$_{\text{NDM-1}}$, *bla*$_{\text{NDM-5}}$, and *bla*$_{\text{NDM-7}}$ on PAK-H surfaces. Each horizontal grey panel represents a PAK-H room. Within each room, the horizontal gridded white lines are the five sampled surfaces. Each vertical white line is one of the collection weeks. Places where the horizontal and vertical white lines intersect represent a sampling. Small circles are *K. pneumoniae* isolates with a *bla*$_{\text{NDM}}$ gene. Large boxes are other bacterial isolates with a *bla*$_{\text{NDM}}$ gene. These boxes are annotated with the specific species identification. Circles and boxes are colored by gene found in the isolate. *bla*$_{\text{NDM-1}}$ is red, *bla*$_{\text{NDM-5}}$ is green, and *bla*$_{\text{NDM-7}}$ is blue.

Source data for all panels is provided in the source data file.

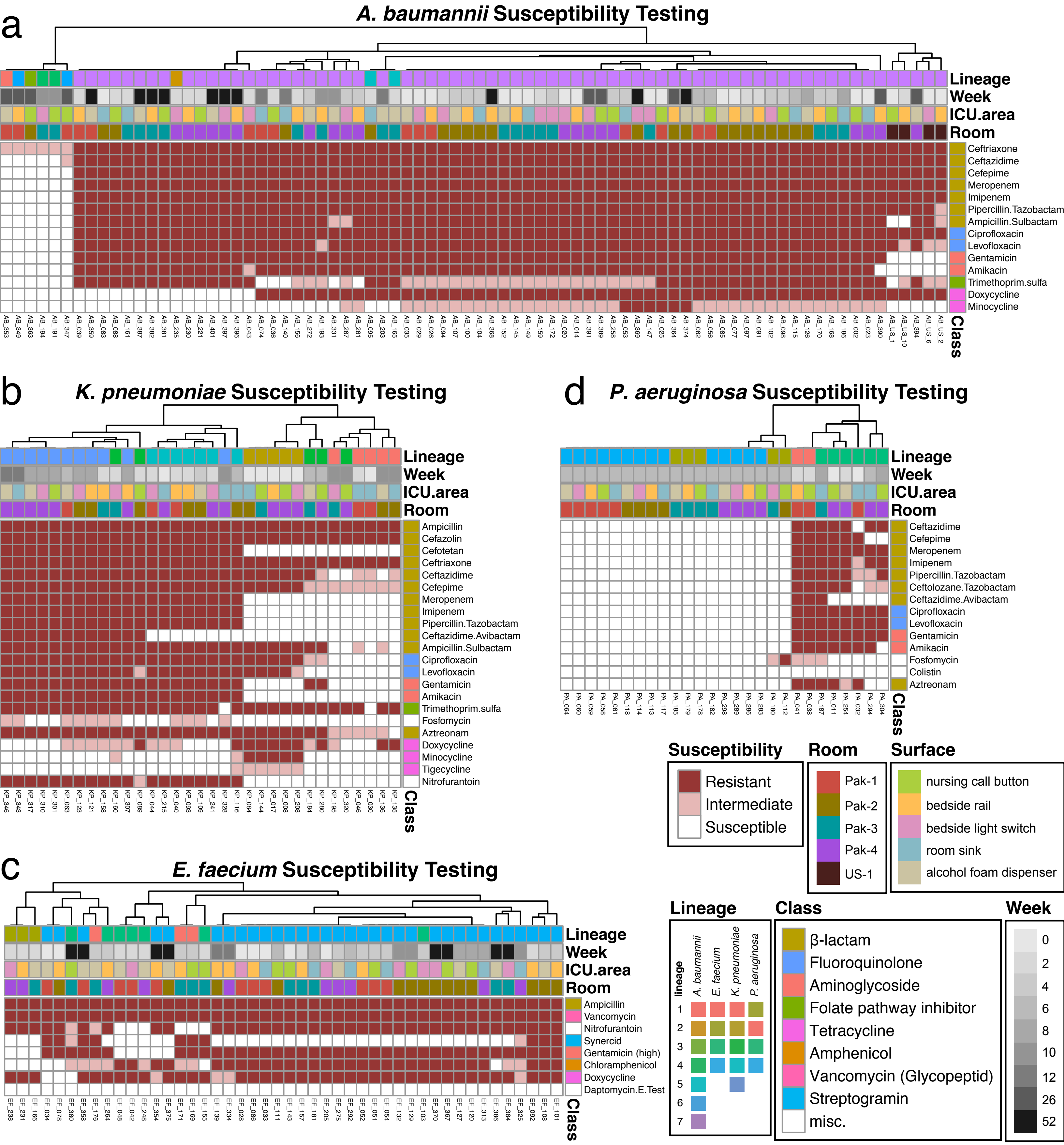
a
A. baumannii Susceptibility Testing
Lineage
Week
ICU.area
Room
Ceftriaxone
Ceftazidime
Cefepime
Meropenem
Imipenem
Pipercillin.Tazobactam
Ampicillin.Sulbactam
Ciprofloxacin
Levofloxacin
Gentamicin
Amikacin
Trimethoprim.sulfa
Doxycycline
Minocycline
Class
b
K. pneumoniae Susceptibility Testing
Lineage
Week
ICU.area
Room
Ampicillin
Cefazolin
Cefotetan
Ceftriaxone
Ceftazidime
Cefepime
Meropenem
Imipenem
Pipercillin.Tazobactam
Ceftazidime.Avibactam
Ampicillin.Sulbactam
Ciprofloxacin
Levofloxacin
Gentamicin
Amikacin
Trimethoprim.sulfa
Fosfomycin
Aztreonam
Doxycycline
Minocycline
Tigecycline
Nitrofurantoin
Class
d
P. aeruginosa Susceptibility Testing
Lineage
Week
ICU.area
Room
Ceftazidime
Cefepime
Meropenem
Imipenem
Pipercillin.Tazobactam
Ceftolozane.Tazobactam
Ceftazidime.Avibactam
Ciprofloxacin
Levofloxacin
Gentamicin
Amikacin
Fosfomycin
Colistin
Aztreonam
Class
c
E. faecium Susceptibility Testing
Lineage
Week
ICU.area
Room
Ampicillin
Vancomycin
Nitrofurantoin
Synercid
Gentamicin (high)
Chloramphenicol
Doxycycline
Daptomycin.E.Test
Class
Susceptibility
Resistant
Intermediate
Susceptible
Room
Pak-1
Pak-2
Pak-3
Pak-4
US-1
Surface
nursing call button
bedside rail
bedside light switch
room sink
alcohol foam dispenser
Lineage
lineage
A. baumannii
E. faecium
K. pneumoniae
P. aeruginosa
1
2
3
4
5
6
7
Class
β-lactam
Fluoroquinolone
Aminoglycoside
Folate pathway inhibitor
Tetracycline
Amphenicol
Vancomycin (Glycopeptid)
Streptogramin
misc.
Week
0
2
4
6
8
10
12
26
52

Supplementary Figure 10: Phenotypic susceptibility results for major species

Phenotypic AST results using CLSI guidelines for **(a)** *A. baumannii*, **(b)** *K. pneumoniae*, **(c)** *E. faecium*, **(d)** *P. aeruginosa*. Antibiotics are grouped by class on the y-axis and individual isolates are hierarchically clustered by their resistance phenotypes on the x-axis. Red squares indicate phenotypic resistance in an isolate, pink represents intermediate resistance, and white represents susceptibility. Colored annotations are added next to antibiotics for drug class and above the charts for hierBAPS lineage, week, surface, and room. Source data for all panels is provided in the source data file.
