# Supplementary material for: Spatiotemporal dynamics of multidrug resistant bacteria on intensive care unit surfaces
Source: Nat Commun. 2019 Oct 8;10:4569. doi: 10.1038/s41467-019-12563-1 (PMC6783542; doi:10.1038/s41467-019-12563-1)
Supplement: Supplementary file 3 — Description of Additional Supplementary Files [file 41467_2019_12563_MOESM3_ESM.pdf]

Description of Additional Supplementary Files

File Name: Supplementary Data 1
Description: **Isolate metadata.** This metadata file includes metadata information for all isolates analyzed in the study. These data include the NCBI submission name, the species identification, the room, week, and ICU.area, and assembly statistics.

File Name: Supplementary Data 2
Description: **Isolate recombination data.** Fastgear recombination data from core genome alignment for A. baumannii, E. faecium, K. pneumoniae, P. aeruginosa, and P. stutzeri.

File Name: Supplementary Data 3
Description: **Pairwise core genome SNP distances.** Pairwise core genome SNP distances for A. baumannii, E. faecium, K. pneumoniae, P. aeruginosa, and P. stutzeri.

File Name: Supplementary Data 4
Description: **SNP based clonal grouping information**. Core genome SNP based clonal groups for A. baumannii and E. faecium.

File Name: Supplementary Data 5
Description: **Spatiotemporal data for SNP based clonal groups.** Observed and expected spatial and temporal distances for A. baumannii and E. faecium core genome SNP based clonal groups.

File Name: Supplementary Data 6
Description: **Variant based clique groupings and spatiotemporal data.** Clique membership and spatiotemporal z-score data for A. baumannii and E. faecium at every variant distance cutoff.

File Name: Supplementary Data 7
Description: **Susceptibility testing.** Susceptibility data for A. baumannii, E. faecium, K. pneumoniae, P. aeruginosa, and P. stutzeri. Breakpoints are given by CLSI guidelines. (coding for the tables is 1 = "Resistant", 0 = "Intermediate", -1 = "Susceptible", -2 = "not tested")

File Name: Supplementary Data 8
Description: **Co-association input tables.** Input tables of species identifications and surface location information for permutation-based co-association testing.

File Name: Supplementary Data 9
Description: **Absolute count co-association test.** Count based co-association test for A. baumannii and E. faecium, including the observed value, the z-score, and the expected co-occurrence distribution.

File Name: Supplementary Data 10
Description: **Frequency based co-association test.** Frequency based co-association test for A. baumannii and E. faecium, including the observed value, the z-score, and the expected co-occurrence distribution.

File Name: Supplementary Software 1
Description: **Example code for variant analysis.** Example R code for identifying cliques at a given cutoff level. Code includes identification of cliques, bootstrap resampling, and z-score testing to relate spatial and temporal data to clique identification.
