# Supplementary material for: Spatiotemporal dynamics of multidrug resistant bacteria on intensive care unit surfaces
Source: Nat Commun. 2019 Oct 8;10:4569. doi: 10.1038/s41467-019-12563-1 (PMC6783542; doi:10.1038/s41467-019-12563-1)
Supplement: Supplementary file 15 — Reporting Summary [file 41467_2019_12563_MOESM15_ESM.pdf]

nature research

Corresponding author(s): Alaric W. D'Souza (during review)
Saadia Andleeb (after publication)
Carey-Ann D. Burnham (after publication)
Gautam Dantas (after publication)

Last updated by author(s): Jul 29, 2019

Reporting Summary

Nature Research wishes to improve the reproducibility of the work that we publish. This form provides structure for consistency and transparency in reporting. For further information on Nature Research policies, see Authors & Referees and the Editorial Policy Checklist .

Statistics

For all statistical analyses, confirm that the following items are present in the figure legend, table legend, main text, or Methods section.

n/a	Confirmed	
☐	☒	The exact sample size (*n*) for each experimental group/condition, given as a discrete number and unit of measurement
☐	☒	A statement on whether measurements were taken from distinct samples or whether the same sample was measured repeatedly
☐	☒	The statistical test(s) used AND whether they are one- or two-sided<br>*Only common tests should be described solely by name; describe more complex techniques in the Methods section.*
☐	☒	A description of all covariates tested
☐	☒	A description of any assumptions or corrections, such as tests of normality and adjustment for multiple comparisons
☐	☒	A full description of the statistical parameters including central tendency (e.g. means) or other basic estimates (e.g. regression coefficient) AND variation (e.g. standard deviation) or associated estimates of uncertainty (e.g. confidence intervals)
☐	☒	For null hypothesis testing, the test statistic (e.g. *F*, *t*, *r*) with confidence intervals, effect sizes, degrees of freedom and *P* value noted<br>*Give P values as exact values whenever suitable.*
☒	☐	For Bayesian analysis, information on the choice of priors and Markov chain Monte Carlo settings
☒	☐	For hierarchical and complex designs, identification of the appropriate level for tests and full reporting of outcomes
☒	☐	Estimates of effect sizes (e.g. Cohen's *d*, Pearson's *r*), indicating how they were calculated

Our web collection on statistics for biologists contains articles on many of the points above.

Software and code

Policy information about availability of computer code

Data collection	No software was used for data collection.
Data analysis	See methods section for detailed description of software and analysis.<br>The reads were demultiplexed by barcode, had adapters removed with Trimmomatic v.36, and contaminating sequences with Deconseq v.4.3. Processed reads were assembled into draft genomes using the de-novo assembler SPAdes v3.11.0. The scaffolds.fasta files were used for all downstream analysis. Assembly statistics on the assemblies was quantified using QUAST v4.5. Prokka v1.12 was ran on the scaffolds file to identify open reading frames > 500 bp in length. MASH was performed against all of the isolate genomes. Isolates that had discrepant analysis were then manually investigated further, by using RNAmmer v1.2 to identify the 16S rRNA sequence, submission of that sequence to the EZ BIoCloud taxonomic database, and finally ANI analysis with the mummer method between the isolate in question and the appropriate type genome (if available) using the JSpecies webserver (http://jspecies.ribohost.com/jspeciesws). All the isolates sequenced in this study were used to construct a Hadamard matrix, representing the product of the average nucleotide identity and percent genome aligned, with the ANIm method from pyANI (https://github.com/widdowquinn/pyani). The matrix was visualized using the python package Seaborn (http://seaborn.pydata.org). The gff files produced from Prokka for A. baumannii, E. faecium, K. pneumoniae, and P. aeruginosa were used to construct a core-genome alignment with Roary v3.8.0 and PRANK v1.0. The recombination purged core genome alignment was used to generate a maximum likelihood tree with RAxML v8.2.11. The output newick file was visualized in iTOL. In silico multilocus sequence typing (MLST) was performed with the MLST program. The sequence type information, week of collection, room of collection, and surface was viewed as a color strip in iTOL. Lineages identified by hierBAPS during fastGEAR were also marked on the trees. Pairwise groupings with 5 or fewer SNPs were imported to Gephi as an unweighted pairwise links table. Gephi's built in modularity analysis was used to isolate perfectly reciprocal groupings. Principal coordinates analysis was done using a gene presence or absence matrix from Roary. Core genes were removed from the matrix and the vegdist function from the Vegan package in R and pcoa function from the ape package in R were used to compute the distance matrix and principal coordinate decomposition respectively for each bacteria. Snippy v4.3.8 (https://github.com/tseemann/snippy) was used to map forward and reverse reads for each isolate to the type strain complete genome assembly (GCF_000746645.1 for A. baumannii and GCF_000174395.2 for E. faecium) and to call variants. Resultant VCF variant files were merged using BCFtools v1.9 with -m all option for multiallelic records.

Merged VCF files were parsed using vcfR. Graph analysis for variant data was conducted using igraph v1.2.4.1. Spatial or temporal distances within clique and expected distributions were calculated by conducting 10,000 permutations of the spatial and temporal distances using the sample function in R v3.53. Acquired ARGs against aminoglycosides, amphenicols, β-lactam, folate pathway inhibitors, fosfomycin, macrolides/lincosamides/streptogramins, quinolones, rifamycin, tetracycline, vancomycin were annotated using the ResFinder BLAST identification program. Presence/absence matrix of ARGs was visualized in pheatmap. Resistance gene network was visualized using Cytoscape v3.4.0. Pairwise BLAST similarity was visualized on the EasyFig v2.2.2 construction by BLASTN similarity between the fasta files.

For manuscripts utilizing custom algorithms or software that are central to the research but not yet described in published literature, software must be made available to editors/reviewers. We strongly encourage code deposition in a community repository (e.g. GitHub). See the Nature Research guidelines for submitting code & software for further information.

Data

Policy information about availability of data

All manuscripts must include a data availability statement. This statement should provide the following information, where applicable:

- Accession codes, unique identifiers, or web links for publicly available datasets
- A list of figures that have associated raw data
- A description of any restrictions on data availability

Assemblies are available from NCBI under BioProject: PRJNA497126. Additional raw data tables, example code for variant analysis, and a graphical depiction of clique identification are included in the zip folder accompanying this submission. A readme file with a list of the files is also in this zip folder.

Field-specific reporting

Please select the one below that is the best fit for your research. If you are not sure, read the appropriate sections before making your selection.

☐ Life sciences ☐ Behavioural & social sciences ☒ Ecological, evolutionary & environmental sciences

For a reference copy of the document with all sections, see nature.com/documents/nr-reporting-summary-flat.pdf

Ecological, evolutionary & environmental sciences study design

All studies must disclose on these points even when the disclosure is negative.

Study description

We longitudinally investigated the burden and transmission dynamics of multidrug resistant bacteria on intensive care unit surfaces from two hospitals in the United States and Pakistan over the course of a year.

Research sample

Phenotypic and genomic analysis was performed on a subset 289 and 6 isolates, respectively from both locations. These bacteria include species which are common nosocomial pathogens, rare opportunistic pathogens, and novel taxa.

Sampling strategy

180 discrete sample collections per hospital were plated onto a suite of selective and differential culture media and had initial species identification assigned by MALDI-TOF MS, which yielded 1,163 bacterial isolates from Pakistan and 23 isolates from the United States.

Data collection

Data was collected by Alaric W. D'Souza, Robert F. Potter, Meghan Wallace, Angela Shupe, Sanket Patel, Xiaoqing Sun, Danish Gul. Intensive care unit rooms were sampled every other week for three months and then at six months and one year after the initial sampling. At each time point, five surfaces were sampled in each patient room. The Eswab collection and transport system (Copan, Murieta, CA) was used to collect all specimens; swabs were moistened prior to sample collection. Two swabs were held together for specimen collection. Specimens collected in Pakistan were shipped to the US site for workup and analysis. Antimicrobial susceptibility testing was performed using Kirby Bauer disk diffusion, interpreted according to CLSI standards. Unique colony morphotypes from the initial swab plates were streaked for isolation on blood agar. After a culture was deemed pure by visual determination, ~10 colonies were suspended in deionized water with a sterile cotton swab. Total genomic DNA was extracted from the suspension using the bacteremia kit. The libraries were pooled and sequenced on a NextSeq HighOutput platform (Illumina) to obtain 2x150 bp reads. For the 11 isolates chosen to be sequenced with Nanopore technology, Genomic DNA was extracted using the Genomic-Tip 500/G (Qiagen) and genomic DNA buffer set (Qiagen) per manufactures instructions. The DNA was converted into a sequencing library on with the Rapid Barcoding Kit (Nanopore, Cambridge, MA, USA) per manufactures instructions and sequenced on the MinION platform. Frozen cultures of A. baumannii ATCC-17978 (17978), A. baumannii ATCC-17978Δpgl, E. faecium TX82, and E. faecium TX5645 were streaked onto tryptic soy agar (Difco, Detroit, MI, USA) and grown overnight at 37 C. Isolated colonies were suspended in tryptic soy broth (Difco, Detroit, MI, USA) supplemented with .5% glucose (MP Biomedicals, Santa Ana, CA, USA) to promote the growth of E. faecium biofilm and quantified for OD600 using a 1:10 dilution. In concordance with previous investigations using respective strains, the A. baumannii isolates were normalized to .05 OD600 and the E. faecium were normalized to .10 OD600. For functional assays. To grow biofilms, 200 µl of each single strain or 100 µl of A. baumannii and 100 µl of E. faecium dual species biofilms were added to tissue culture treated 96 well polystyrene microtiter plates (Sigma Aldrich, St. Louis, MO, USA) in triplicate. We additionally plated cell-free controls to ensure that no contamination occurred and to subtract out background absorbance reading. After pipetting, the plates were gently pipetted up and down to ensure that the strains mixed thoroughly. The plates were covered with breath ez membrane (Diversified Biotech, Dedham, MA, USA) and grown on the benchtop at approximately 22 Celsius for 16 hours. Following a growth period, the biofilm plates had planktonic cells removed by washing thoroughly with 250 µl sterile phosphate buffered saline (PBS) (Thermo Fisher Scientific, Waltham, MA, USA) three times. To obtain the total biofilm biomass, the washed biofilms were fixed with 250 µl bouin's solution (Sigma Aldrich) at 22 ° Celsius on the benchtop for 30 minutes. The fixative was washed three times with 200 µl sterile PBS three times and then stained with 250 µl .01% crystal violet (Sigma Aldrich) in water for 30 minutes at 22 °Celsius on the bench. Finally, the unstained crystal violet was removed by washing three times with PBS and then the biomass was solubilized with 250 µl of 100% ethanol (Sigma Aldrich). The amount of biofilm biomass was quantified using nm absorbance with a Synergy H1(BioTek) spectrophotometry machine. All raw absorbance values were adjusted by removing the

background values obtained from the cell-free TSB controls. For quantification of total viable cells in the biofilm, the biofilms were formed as previously described. After 16 hours growth at 22 ° Celsius, planktonic cells were removed by washing thoroughly with 250 µl PBS. The XTT cell viability kit (Cell Signaling Technologies, Danvers, MA, USA) was then performed according to manufacturer's instructions. The plates were read in the Synergy H1 spectrophotometry machine after 5-hour incubation in the dark. For the crystal violet and XTT reduction assays, the biofilm synergy scores were calculated as previously reported for dual species biofilms. For each pairwise comparison, the synergy scores were reported as the difference between the average plus standard deviation for the single species biofilm and average minus standard deviation of the dual species biofilm.
Please see manuscript for additional details.

Timing and spatial scale

Data was collected from March 2016 to March 2017. This long time scale was chosen to assess longitudinal persistence of bacteria on the surfaces. We included a mix of higher resolution (every 2 weeks) and lower resolution (3 month and 6 month) intervals for our collections. Samples were collected from a tertiary care hospital and Pakistan and a tertiary care hospital in the United States. All spatial comparisons are made on the within hospital scale.

Data exclusions

We recovered 1163 bacterial isolates from the hospital surfaces in PAK-H and predicted their species identities by MALDI-TOF MS. We chose a subset of 289 unique isolates for phenotypic and genomic analysis, using the criterion of only considering a single isolate per unique MALDI-TOF MS identified species per culture condition per surface per time-point.

Reproducibility

We included a matched tertiary care hospital in the USA (USA-H) as a comparison group in this study. Wet lab experiments were completed in triplicate and repeated each time. Analysis was done in comparison to null models generated by random permutation.

Randomization

This is not relevant to the study because we collected data directly from surfaces. Since we did not infect surfaces in the study, there was nothing to randomize.

Blinding

Blinding was not relevant to this study. We did not have experimental groups for the study since we were collecting bacteria from hospital surfaces. Additionally, the collections were wildly different between the two countries in terms of the numbers of bacteria collected.

Did the study involve field work? ☐ Yes ☒ No

Reporting for specific materials, systems and methods

We require information from authors about some types of materials, experimental systems and methods used in many studies. Here, indicate whether each material, system or method listed is relevant to your study. If you are not sure if a list item applies to your research, read the appropriate section before selecting a response.

Materials & experimental systems

n/a	Involved in the study
☒	☐ Antibodies
☒	☐ Eukaryotic cell lines
☒	☐ Palaeontology
☒	☐ Animals and other organisms
☒	☐ Human research participants
☒	☐ Clinical data

Methods

n/a	Involved in the study
☒	☐ ChIP-seq
☒	☐ Flow cytometry
☒	☐ MRI-based neuroimaging
